# Effectiveness of Smartphone App–Based Interactive Management on Glycemic Control in Chinese Patients With Poorly Controlled Diabetes: Randomized Controlled Trial

**DOI:** 10.2196/15401

**Published:** 2019-12-09

**Authors:** Lei Zhang, Xingxing He, Yun Shen, Haoyong Yu, Jiemin Pan, Wei Zhu, Jian Zhou, Yuqian Bao

**Affiliations:** 1 Department of Endocrinology and Metabolism, Shanghai Clinical Center for Diabetes, Shanghai Diabetes Institute, Shanghai Key Laboratory of Diabetes Mellitus, Shanghai Jiao Tong University Affiliated Sixth People’s Hospital Shanghai China

**Keywords:** app, self-management, interactive management, guidance, glycated hemoglobin A1c, diabetes

## Abstract

**Background:**

In recent years, the rapid development of mobile medical technology has provided multiple ways for the long-term management of chronic diseases, especially diabetes. As a new type of management model, smartphone apps are global, convenient, cheap, and interactive. Although apps were proved to be more effective at glycemic control, compared with traditional computer- and Web-based telemedicine technologies, how to gain a further and sustained improvement is still being explored.

**Objective:**

The objective of this study was to investigate the effectiveness of an app-based interactive management model by a professional health care team on glycemic control in Chinese patients with poorly controlled diabetes.

**Methods:**

This study was a 6-month long, single-center, prospective randomized controlled trial. A total of 276 type 1 or type 2 diabetes patients were enrolled and randomized to the control group (group A), app self-management group (group B), and app interactive management group (group C) in a 1:1:1 ratio. The primary outcome was the change in glycated hemoglobin (HbA_1c_) level. Missing data were handled by multiple imputation.

**Results:**

At months 3 and 6, all 3 groups showed significant decreases in HbA_1c_ levels (all *P*<.05). Patients in the app interactive management group had a significantly lower HbA_1c_level than those in the app self-management group at 6 months (*P*=.04). The average HbA_1c_ reduction in the app interactive management group was larger than that in the app self-management and control groups at both months 3 and 6 (all *P*<.05). However, no differences in HbA_1c_ reduction were observed between the app self-management and control groups at both months 3 and 6 (both *P*>.05). Multivariate line regression analyses also showed that the app interactive management group was associated with the larger reduction of HbA_1c_ compared with groups A and B at both months 3 and 6 (all *P*>.05). In addition, the app interactive management group had better control of triglyceride and high-density lipoprotein cholesterol levels at both months 3 and 6 compared with baseline (both *P*<.05).

**Conclusions:**

In Chinese patients with poorly controlled diabetes, it was difficult to achieve long-term effective glucose improvement by using app self-management alone, but combining it with interactive management can help achieve rapid and sustained glycemic control.

**Trial Registration:**

ClinicalTrials.gov NCT02589730; https://clinicaltrials.gov/ct2/show/NCT02589730.

## Introduction

### Effectiveness of Smartphone Apps on Glycemic Control

The rapid development of mobile medical technology has provided multiple ways for the long-term management of chronic diseases, especially diabetes [1]. As a new type of management model, smartphone apps are global, convenient, cheap, and interactive. Compared with traditional computer- and Web-based telemedicine technologies [2,3], apps may have unique advantages in diabetes management [4-6]. Recent studies have proved that apps are effective at improving glycated hemoglobin (HbA_1c_) levels and could be considered as an adjuvant intervention to diabetes management [7-11].

### Demand for Further and Sustained Glycemic Control

China has the world’s largest population with diabetes [12] but a shortage of physicians. Under such special national conditions, scientific and effective diabetes management models are urgently needed for better outcomes. Although apps were proved to be effective at glycemic control, how to gain a further and sustained improvement is still being explored. Welltang, designed in Chinese language, is the most widely used app for diabetes management in China. It was previously reported in a 3-month randomized controlled trial that diabetes patients using the Welltang app achieved statistically significant improvements in HbA_1c_, with an average decrease of 1.95%, whereas the reduction in HbA_1c_ was just 0.79% in the control group [9]. However, the duration of this previous study was relatively short, and there was a lack of a professional health team to provide interactions with patients beyond medical adjustment, such as diet, exercise, and diabetes education.

### Aim of This Study

Therefore, this single-center, open-labeled, prospective randomized controlled trial was conducted in Chinese patients with poorly controlled diabetes, including both type 1 and type 2 diabetes, to investigate the effectiveness of an app-based interactive management model on glycemic control and to explore an individualized method for diabetes management in China.

## Methods

### Trial Design

This study was a 6-month long, single-center, open-labeled, prospective randomized controlled trial. Participants were recruited from the outpatient clinic of the Department of Endocrinology and Metabolism of Shanghai Jiao Tong University Affiliated Sixth People’s Hospital from July 2015 to February 2016. According to the type of diabetes mellitus (type 1 or 2 diabetes), the stratified randomization method was used to generate a random number table, and participants were randomized into 3 groups in a 1:1:1 ratio: group A (control group), group B (smartphone app self-management group), and group C (smartphone app interactive management group). This study was registered at ClinicalTrials.gov, number NCT02589730.

### Study Patients

Outpatients diagnosed with type 1 or 2 diabetes (aged 18-65 years) were enrolled according to the 2010 American Diabetes Association criteria [13], with a duration ≥6 months and HbA_1c_ ≥8% within 3 months before enrollment. To be eligible, patients had to be able to use a smartphone, be willing and able to perform daily self-monitoring of blood glucose (SMBG), and be willing and able to visit a physician at months 3 and 6.

Patients were excluded according to the following criteria: (1) insulin pump users; (2) pregnant or plan to be pregnant during the study period; (3) excessive drinking or drug users; (4) used drugs that might affect blood sugar in the 3 months before enrollment, such as glucocorticoids and thyroid hormones; (5) psychotic and were receiving treatment; (6) severe complications or systemic diseases; (7) experienced cardio- or cerebrovascular events in the 6 months before enrollment; (8) severe hearing or visual impairment; (9) unable to access the Web or unable to learn to use the app on the smartphone; and (10) unsuitable for the study according to the judgment of the researchers.

The study was approved by the Ethics Committee of the Shanghai Jiao Tong University Affiliated Sixth People’s Hospital and conformed to the provisions of the Declaration of Helsinki. All participants provided written informed consent before enrollment.

### Interventions

The smartphone-based diabetes management platform in this study was Welltang, which was designed by Shanghai Geping Information and Technique Company Ltd, and it was used by both patients and clinicians. For patients, Welltang mainly comprises 4 parts: education, self-management (including records of SMBG, diet, exercise, medication, body weight, and other diabetes data), patient community, and communication between patients and clinicians. For clinicians, Welltang mainly provided the real-time uploading of data from patients.

In group A, patients received usual care and did not install Welltang on their smartphone. They learned diabetes-related knowledge and skills by self-learning and summarizing, and they adopted lifestyles and behaviors voluntarily.

In group B, each patient was requested to install Welltang on their smartphone. They learned diabetes-related knowledge and skills by using the app, including glycemic control, diet, exercise, medication, and the use of insulin. There was no other staff involved in the care of this group, except for 1 clinician.

In group C, besides app self-management, patients received interactive management online (service for stable glucose×180 days). After randomization, a third-party professional diabetes health care team comprising 1 dietician and 1 health manager conducted interactive management with patients through the Welltang platform. The dietician was responsible for daily dietary guidance and the health manager for comprehensive interventions, such as exercise, glucose monitoring, and diabetes education based on the data uploaded by the patients. The service had a standardized operation process. During the first month, centralized management was conducted for reasons of poor glycemic control to help patients develop good habits of glucose control. Afterward, patients were evaluated monthly. When blood glucose (BG) fluctuated greatly, the causes of fluctuation were analyzed to improve glycemic control. When the patients achieved certain improvements and gained inertia, reminders were provided promptly to prevent large fluctuations. Finally, this team also assisted patients with achieving a relatively stable period of managing diabetes by themselves. During the service, a glucose control report was generated every week, and appropriate suggestions were provided according to the report.

After enrollment, each patient was provided an optimal glucose control target according to the *Guidelines for the Prevention and Treatment of Type 2 Diabetes in China (2013 Edition)* [14] and received basic diabetes education, including diet control, adequate exercise, SMBG, and regular follow-up. All patients could contact clinicians by telephone during the follow-up, but those who had installed the app were encouraged to contact clinicians online. Clinicians were blind to the patients’ groups. The dietician and health manager could view all patients’ clinical variables and provide real-time interventions and adjustments on the basis of these data.

Each patient was equipped with a designated BG meter and an adequate number of test strips. Patients in the app self-management and app interactive management groups were asked to regularly record the glucose results in Welltang; patients in the control group were asked to record their glucose results in a log book. The frequency of SMBG that was required was at least 6 times a week, without a maximum limit. Test trips were provided by the investigators for free.

### Data Collection

The follow-up duration was 6 months. At baseline, all patients were asked to complete a questionnaire about demographic characteristics, personal history, and medical history. Anthropometric and clinical measurements were collected from all patients at baseline and at months 3 and 6 after the intervention.

Body mass index (BMI) was calculated as BMI=weight (kg)/height^2^ (m^2^). Blood pressure was measured with a mercury sphygmomanometer after the subject had rested for at least 10 min. Waist circumference (W) was measured midway between the lowest rib and the iliac crest with the subject in the standing position.

Venous blood was collected in the morning after a 10-hour overnight fast. The levels of fasting plasma glucose (FPG), HbA_1c_, total cholesterol (TC), triglyceride (TG), high-density lipoprotein cholesterol (HDL-c), low-density lipoprotein cholesterol (LDL-c), alanine aminotransferase, aspartate aminotransferase, gamma-glutamyl transpeptidase, creatinine, uric acid, and albumin-to-creatinine ratio were assessed with standard methods, as described previously [15].

### Adverse Event

In this study, the major adverse event was hypoglycemia. Hypoglycemia was defined as BG ≤3.9 mmol/L. Each patient had received related education and treatment measures after enrollment and could call physicians when they needed. All adverse events were recorded.

### Outcomes

The primary outcome was glucose control, including the changes (from baseline to months 3 and 6) in the HbA_1c_ level. The secondary outcomes included the changes in FPG, body weight, and lipids.

### Sample Size

In a small-sample (n=9) observational study conducted before this trial, the reductions of HbA_1c_ in the control, app self-management, and app interactive management groups after 3 months of follow-up were 0.9% (SD 0.9%), 1.3% (SD 1.1%), and 1.6% (SD 1.3%), respectively. On the basis of these results from the small-sample observational study, with alpha=.05 and beta=.10, we calculated a required sample size of 65 patients per group by PASS 11.0 (NCSS LLC) software. Considering a dropout rate of 20%, a sample size of 78 patients per group was required.

### Statistical Analysis

The statistical analysis was performed based on the intention-to-treat principle. Missing data were handled by multiple imputation [16,17]. The R multivariate imputation by chained equation package was used to impute 5 sets of complete dataset with 50 iterations per imputation. Predictive mean matching was used for continuous variables. All findings are presented based on multiply imputed data, unless otherwise indicated.

The data are reported as the mean (SD), unless otherwise stated. Intergroup comparisons were conducted with the unpaired Student *t* test, Kruskal-Wallis test, and chi-square test for normally distributed data, skewed data, and categorical variables, respectively. The paired Student *t* test was used to evaluate the differences in continuous variables from baseline to months 3 and 6 of follow-up in each group. The multiple linear regression analyses were applied to explore the associations of HbA_1c_ level reduction and different models of diabetes management. All data analyses were conducted by SPSS version 19.0 (SPSS). A 2-tailed *P* value of <.05 was considered indicative of a statistically significant difference.

## Results

### Baseline Characteristics of Study Participants

**Figure 1:**
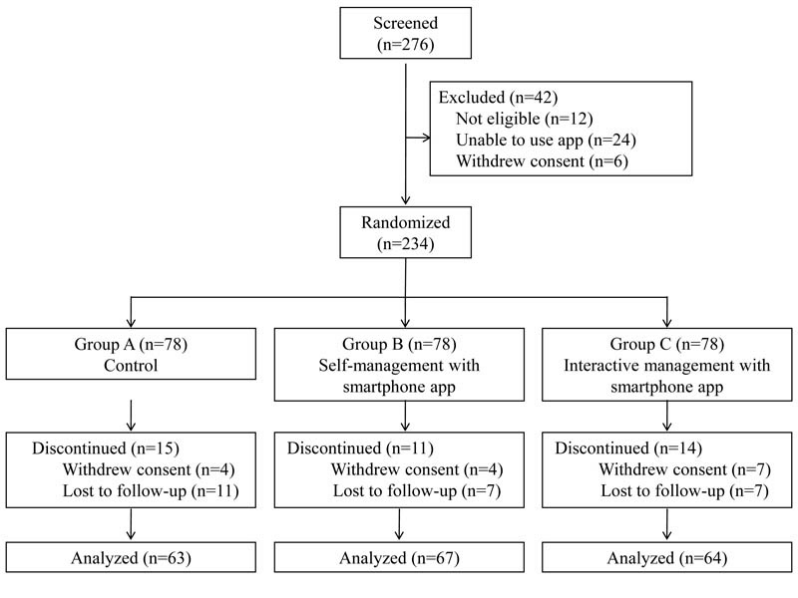
Study design and participant flow diagram.

Of the 276 participants who underwent screening in this study, 234 were enrolled and randomized to groups A (n=78), B (n=78), or C (n=78). Of these, a total of 209 (209/234, 89.3%) completed the third month visit and 194 (194/234, 82.9%) completed the sixth month visit. After randomization, 15 patients from group A, 11 from group B, and 14 from group C discontinued the study because they either withdrew their consent or were lost to follow-up (Figure 1).

The demographic and baseline clinical characteristics of the 3 groups are presented in Table 1. Among the patients enrolled, the mean age was 53 (SD 11) years, mean diabetes duration was 11.3 (SD 6.1) years, 8.9% (21/234) had type 1 diabetes, 38.0% (89/234) were female, 46.2% (108/234) had junior college or higher education, and 58.1% (136/234) were working. The average BMI at baseline was 25.03 (SD 3.36) kg/m^2^, and the average FPG and HbA_1c_ levels were 10.02 (SD 3.03) mmol/L and 9.45% (SD 1.36%), respectively.

**Table 1:** Baseline characteristics of study participants of the 3 groups.

Baseline characteristics		Group A^a^	Group B^b^	Group C^c^
Gender (men/women), n		78 (49/29)	78 (50/28)	78(46/32)
Age (years), mean (SD)		55 (11)	52 (10)	52 (12)
Diabetes duration (years), mean (SD)		12.7 (7.1)	11.2 (5.6)	10.1 (5.5)
Type 1 diabetes, n (%)		7 (9)	7 (9)	7 (9)
Body mass index (kg/m^2^), mean (SD)		24.63 (2.79)	25.36 (3.32)	25.09 (3.88)
Waist circumference (cm), mean (SD)		85.2 (7.7)	87.2 (8.9)	87.0 (10.6)
**Blood pressure (mmHg), mean (SD)**				
	Systolic	127 (13)	127 (12)	127 (15)
	Diastolic	79 (9)	81 (9)	80 (7)
Fasting plasma glucose (mmol/L), mean (SD)		9.86 (3.17)	10.06 (3.03)	10.14 (2.92)
Glycated hemoglobin (%), mean (SD)		9.27 (1.25)	9.46 (1.18)	9.58 (1.62)
Total cholesterol (mmol/L), mean (SD)		4.92 (1.10)	4.65 (0.87)	5.01 (0.79)
Triglyceride (mmol/L), mean (SD)		1.84 (1.05)	1.87 (1.14)	2.39 (1.63)
**Lipoprotein density (mmol/L), mean (SD)**				
	High	1.11 (0.27)	1.08 (0.27)	1.07 (0.28)
	Low	2.97 (0.94)	2.79 (0.75)	2.90 (0.79)
**Aminotransferase (U/L), mean (SD)**				
	Alanine	27 (19)	28 (24)	35 (28)
	Aspartate	21 (8)	22 (12)	25 (15)
Gamma-glutamyl transpeptidase (U/L), mean (SD)		36 (21)	39 (25)	40 (23)
Creatinine (µmol/L), mean (SD)		65 (16)	64 (13)	62 (13)
Uric acid (µmol/L), mean (SD)		303 (67)	305 (78)	305 (78)
**Education, n (%)**				
	Junior college or higher	32 (41)	38 (48.)	38 (48)
	High school	27 (34)	25 (32)	28 (35)
	Primary school or less	19 (24)	15 (19)	12 (15)
Working, n (%)		39 (50)	50 (64)	47 (60)
Insulin, n (%)		33 (42)	33 (42)	41 (52)
Hypertension, n (%)		29 (37)	23 (29)	28 (35)
Current smoker, n (%)		18 (23)	13 (16)	14 (17)
Current drinker, n (%)		13 (16)	6 (7)	11 (14)

^a^Group A: control group.

^b^Group B: app self-management group.

^c^Group C: app interactive management group.

### Primary Study Outcomes: The Change in the Glycated Hemoglobin Level

Only a significant difference in HbA_1c_ levels was observed between groups A and C at baseline (average change in HbA_1c_ in groups A vs group C: 9.14% [SD 1.13%] vs 9.60% [SD 1.44%]; **t*_125_*=−1.995; *P*=.048). Compared with baseline, all 3 groups showed significant decreases in HbA_1c_ levels at both months 3 and 6 (all *P*<.001). At month 3, the mean HbA_1c_ levels in groups A, B, and C were 8.12% (SD 1.21%), 7.94% (SD 1.35%), and 7.59% (SD 0.95%), respectively. Intergroup comparisons only showed a significant difference between groups A and C (**t*_125_*=2.732; *P*=.007). At month 6, the mean HbA_1c_ levels in groups A, B, and C were 7.80% (SD 1.14%), 8.04% (SD 1.38%), and 7.57% (SD 1.18%), respectively. Intergroup comparisons showed that HbA_1c_ levels in group C were significantly lower than those in group B (**t*_129_*=2.072; *P*=.04; Multimedia Appendix 1).

To further compare the improvement of glycemic control in the 3 different management groups, we analyzed the mean HbA_1c_ level reduction from baseline to months 3 and 6. We found that at the end of month 3, the average decrease in HbA_1c_ levels in group C was larger than that in groups A and B (group C vs group A: −2.00% [SD 1.45%] vs −1.01% [SD 1.42%]; **t*_125_*=3.876; *P*<.001; group C vs group B: −2.00% [SD 1.45%] vs −1.46% [SD 1.52%]; **t*_129_*=2.091; *P=*.04). At month 6, the HbA_1c_ reduction in group C was still significantly larger than that in groups A and B (group C vs group A: −2.03% [SD 1.56%] vs −1.34% [SD 1.50%]; **t*_125_*=2.515; *P*=.01; group C vs group B: −2.03% [SD 1.56%] vs −1.37% [SD 1.48%]; **t*_129_*=2.488; *P*=.01). However, no differences were observed between groups B and A at both months 3 and 6 (both *P*>.05; Multimedia Appendix 1).

Multivariate linear regression analyses defined HbA_1c_ reduction as a dependent variable and the group A or group B as reference. After adjustment of age, gender, W, BMI, diabetes duration, systolic blood pressure, diastolic blood pressure, TC, TG, HDL-c, and LDL-c, the results showed whether at months 3 or 6, group C was associated with the larger reduction of HbA_1c_, compared with groups A and B (all *P*<.05). But this association was not significant between groups B and A at both months 3 and 6 (both *P*>.05; Table 2).

**Table 2:** Multivariate linear regression analyses of glycated hemoglobin reduction.^a^

Diabetes management models			Beta value	SE	Standardized beta (95% CI)	*P* value
**At 3 months**						
	**Model 1**					
		Group A^b^ (reference)	—^c^	—	—	—
		Group B^d^	−.242	0.259	−.076 (−0.752 to −0.268)	.35
		Group C^e^	−.826	0.260	−.258 (−1.339 to −0.314)	.002
	**Model 2**					
		Group B (reference)	—	—	—	—
		Group C	−.585	0.250	−.182 (−1.078 to −0.092)	.02
**At 6 months**						
	**Model 3**					
		Group A (reference)	—	—	—	—
		Group B	.097	0.267	.030 (−0.431 to 0.625)	.72
		Group C	−.553	0.269	−.169 (−1.083 to −0.023)	.04
	**Model 4**					
		Group B (reference)	—	—	—	—
		Group C	−.650	0.258	−.199 (−1.159 to −0.140)	.01

^a^Independent variables originally included the following: age, gender, W, BMI, diabetes duration, systolic blood pressure, diastolic blood pressure, TC, TG, HDL-c, and LDL-c.

^b^Group A: control group.

^c^Do not have statistical data.

^d^Group B: app self-management group.

^e^Group C: app interactive management group.

### Secondary Study Outcomes: Fasting Plasma Glucose, Body Weight, and Lipid Levels

At the end of month 3, group C experienced significant improvement in FPG levels (mean FPG at month 3 vs baseline: 8.12 [SD 2.07] mmol/L vs 9.91 [SD 2.93] mmol/L; **t*_63_*=4.251; *P*<.001), whereas the difference was not significant in both groups A and B (*P=*.86 and *P*=.21, respectively). Intergroup comparisons found that the FPG level in group C was lower than that in groups A and B (mean FPG in group C vs group A: 8.12 [SD 2.07] mmol/L vs 9.10 [SD 2.78] mmol/L; **t*_125_*=2.235; *P*=.03; group C vs group B: 8.12 [SD 2.07] mmol/L vs 9.14 [SD 2.61] mmol/L; **t*_129_*=2.469; *P*=.02). At month 6, still only group C had a significantly lower FPG level than that at baseline (mean FPG at month 6 vs baseline: 7.87 [SD 2.07] mmol/L vs 9.91 [SD 2.93] mmol/L; **t*_63_*=4.920; *P*<.001). The FPG level in group C was still significantly lower than that in both groups B and A at month 6 (mean FPG in group C vs group A: 7.87 [SD 2.07] mmol/L vs 8.91 [SD 2.81] mmol/L; **t*_125_*=2.372; *P*=.02; group C vs group B: 7.87 [SD 2.07] mmol/L vs 9.08 [SD 2.91] mmol/L; **t*_129_*=2.731; *P*=.007). No differences were observed between groups B and A at both months 3 and 6 (both *P*>.05).

All 3 groups had better control of HDL-c levels at both months 3 and 6 (all *P*<.05). Group C had better control of TG and HDL-c levels at both months 3 and 6 compared with those at baseline (all *P*<.05). There were no significant differences among the 3 groups for body weight, TC, TG, and LDL-c at both months 3 and 6 (all *P*>.05; Multimedia Appendix 2).

### Blood Glucose Test Rate, Frequency of App Usage, and Guiding Time

No significant differences in the BG test rate were reported among the 3 groups at both months 3 and 6. After 3 months of follow-up, the average weekly frequencies of BG test rates in groups A, B, and C were 10.9 (SD 7.8) times, 10.1 (SD 9.5) times, and 11.1 (SD 8.9) times, respectively, and were 10.5 (SD 7.5) times, 10.1 (SD 9.3) times, and 9.4 (SD 6.9) times at the end of month 6, respectively.

The frequencies of app usage in groups B and C were 10.7 (SD 9.5) times per week in group B and 11.1 (SD 7.3) times per week in group C, and the difference was not significant (*P*=.83).

In group C, each patient received an average of 30.5 (SD 3.6) times of interactive management during the whole follow-up, with an average guiding time of 458 (SD 54) min.

### Adverse Events

The major adverse event in this study was hypoglycemia. Hypoglycemia was defined as a BG ≤3.9 mmol/L. At the end of month 6, the frequency of hypoglycemia was similar in the 3 groups (average frequency in group A: 6.9 [SD 6.3] times per person, group B: 6.7 [SD 6.2] times per person, and group C: 5.7 [SD 4.9] times per person). During the follow-up, none of the patients dropped out because of hypoglycemia.

## Discussion

### Summary of Principal Findings

This study found that the app interactive management group had a greater improvement in HbA_1c_ levels compared with both the app self-management and control groups. However, no significant differences were observed between the app self-management and control groups in the reduction of HbA_1c_ levels, indicating that when using the app for diabetes management, only self-management is not enough, and the combination with interactive management can provide better glycemic control and a longer effect.

Recent studies have shown that a smartphone-based app was a feasible and an effective tool for diabetes management [4,7,18-20]. Waki et al [21] used an app named *DialBetics* in a 3-month randomized study, which enrolled 54 patients with type 2 diabetes, and found that HbA_1c_ decreased with an average of 0.4% (from 7.1% [SD 1.0%] to 6.7% [SD 0.7%]) in the DialBetics group, whereas it increased with an average of 0.1% (from 7.0% [SD 0.9%] to 7.1% [SD 1.1%]) in the non-DialBetics group. Another randomized trial conducted in 185 Chinese patients with type 2 diabetes also found that the implementation of the app *Diabetes-Carer* was effective at improving the proportion of type 2 diabetes patients with a HbA_1c_ <7% [8]. In this study, consistent with the previous studies, the app interactive management group had a better improvement in HbA_1c_ levels compared with both the app self-management and control groups after 6 months of follow-up, however, no significant differences were observed between the app self-management and control groups at both months 3 and 6. These results indicated that the effect of app self-management may not be significantly superior to usual care. Limited by the large number of diabetes patients but a shortage of medical staff in China, clinicians and nurses always lack time to provide normative and continuous management for diabetes patients. In addition, test strips are not covered by most health care insurances, which may cause a heavy financial burden for most of the patients. Considering these factors, this study also provided equally basic diabetes education to the control group and provided adequate test strips to all patients for free. Therefore, the benefits of glycemic control may be because of the management in diabetes patients themselves rather than the unique role of the app. In addition, the better control of BG in the app interactive management group than the control and app self-management groups might be able to reflect the importance of guidance rather than the provision of free test strips only. With better control of BG, patient’s health and quality of life might also improve.

In most of the previous diabetes management–related apps, clinicians could view the data and queries uploaded by patients on the app platform; however, the interventions were limited to drug adjustments in most of the cases [5,17,22]. However, lifestyle adjustments, including healthy diet and regular exercise, are equally important for glycemic control and prevention of diabetes-related complications [23-26]. Owing to a shortage of medical staff in China, it is very difficult to provide comprehensive and effective guidance to every diabetes patient. Therefore, based on the use of Welltang, we introduced a third-party professional health care team to actively interact with patients online to provide a full range of guidance. The results showed that after 6 months of follow-up, HbA_1c_ levels in group C had decreased by an average of 2.03%, which was significantly higher than that in group B, with an average decrease of 1.37%. In addition, compared with that at baseline, group C achieved better control of TG and HDL-c levels at both months 3 and 6, whereas the differences were not significant in group B. These findings indicated that app self-management combined with a professional health team could achieve better glycemic and lipid control. The interactive management model may be one of the models that truly promote diabetes management in the future.

In addition, we also found that in the third month of follow-up, all 3 groups showed significant decreases in HbA_1c_ levels. However, in the following 3 months, there was no significant improvement in HbA_1c_ levels. In group C, the HbA_1c_ level decreased an average of 0.03%, whereas it increased an average of 0.09% in group B. Interestingly, in group A, the HbA_1c_ level decreased an average of 0.33%. Our results indicated that the app was able to achieve a quick and effective improvement in HbA_1c_ levels in a short time. However, in the following 3 months, the effects appear to have a *platform period*, and group A even had a nearly equal effect compared with group B. Similar phenomena have been observed in previous studies [8,27]. Interestingly, in a review of diabetes education, researchers also found that group and individual education had an equal impact on HbA_1c_ at 12 to 18 months [28]. Patients’ inertia after receiving management for a relatively long time and different glycemic control targets from clinicians may contribute to this phenomenon.

In this trial, each participant was equipped with a BG meter and sufficient test strips. During the follow-up, the frequency of SMBG in the 3 groups was almost the same, which was approximately 11 times a week. Web-based care management in patients with poorly controlled diabetes showed that a larger number of website SMBG data uploads was associated with a larger decline in HbA_1c_ [29]. Another study *Engaging and Motivating Patients Online With Enhanced Resources for Diabetes*, which was aimed to evaluate an online disease management system supporting patients with uncontrolled type 2 diabetes, also demonstrated that participants who tested their glucose at home and uploaded their results more often were more likely to have improved at 6 and 12 months than those who did not [2]. In this study, group C had a full-time third-party diabetes health team reviewing all patients’ uploaded data every week and making recommendations about diet and exercise management, which could help further improve glycemic control.

### Limitations

There are still some limitations in this study. First, it was a single-center study, and the follow-up duration was only 6 months. Considering the chronic and long-term characteristics of diabetes, the study was unable to assess the long-term effects of a smartphone-based app on diabetes management; further multicenter studies with longer follow-up are needed to evaluate the long-term benefits of smartphone apps on diabetes management. Second, owing to the trial requiring that patients be able to use a smartphone phone, only those aged less than 65 years were enrolled. The results may not entirely reflect the characteristics of all diabetes populations. Third, the sample of type 1 diabetes patients in this study was small; further studies in different types of diabetes are needed to provide more detailed results. Finally, this study also provided equally basic diabetes education and adequate test strips to the control group, which might cause the control condition to not be a true treatment as usual condition.

### Conclusions

In conclusion, we found that the effect of app self-management alone in Chinese diabetes patients with poor glycemic control was not superior to routine management, and the combination with interactive management can help achieve rapid and sustained glycemic control.

## Appendix

Multimedia Appendix 1 (A) Glycated hemoglobin (HbA_1c_) levels during 6 months in the 3 groups. (B) The average reductions of HbA_1c_ levels during 6 months in the 3 groups. Group A: control group, Group B: app self-management group, and Group C: app interactive management group. HbA_1c_: glycated hemoglobin.

Multimedia Appendix 2 Levels of fasting plasma glucose, Body Weight and Lipids during the follow-up.

Multimedia Appendix 3 CONSORT-EHEALTH checklist (V1.6.1).

## Abbreviations

BG: blood glucose
BMI: body mass index
FPG: fasting plasma glucose
HbA_1c_: glycated hemoglobin
HDL-c: high-density lipoprotein cholesterol
LDL-c: low-density lipoprotein cholesterol
SMBG: self-monitoring of blood glucose
TC: total cholesterol
TG: triglyceride
W: waist circumference
