# Supplementary figures and images for: Effectiveness of Smartphone App–Based Interactive Management on Glycemic Control in Chinese Patients With Poorly Controlled Diabetes: Randomized Controlled Trial
Source: J Med Internet Res. 2019 Dec 9;21(12):e15401. doi: 10.2196/15401 (PMC6928697; doi:10.2196/15401)

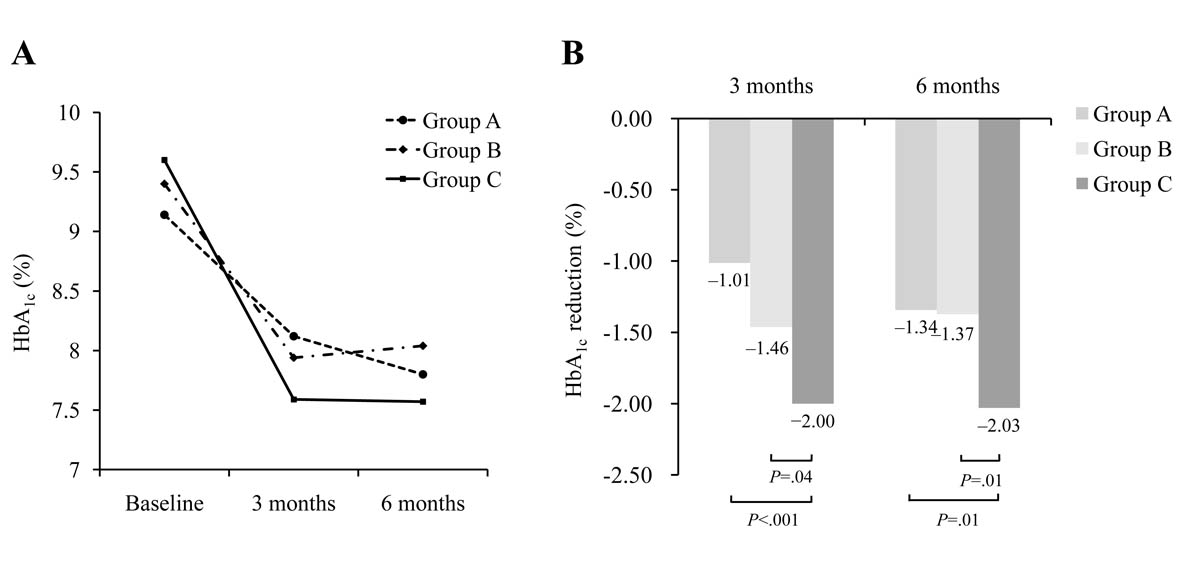

Supplement: Multimedia Appendix 1 [file jmir_v21i12e15401_app1.png]
