# Supplementary material for: Effectiveness of Smartphone App–Based Interactive Management on Glycemic Control in Chinese Patients With Poorly Controlled Diabetes: Randomized Controlled Trial
Source: J Med Internet Res. 2019 Dec 9;21(12):e15401. doi: 10.2196/15401 (PMC6928697; doi:10.2196/15401)
Supplement: Multimedia Appendix 2 [file jmir_v21i12e15401_app2.docx]

**Multimedia Appendix 1.** **Levels of FPG, Body Weight and Lipids during the follow-up**

|  | Group A | Group B | Group C | *P* (Group A *vs* B) | *P* (Group A *vs* C) | *P* (Group B *vs* C) |
| --- | --- | --- | --- | --- | --- | --- |
| FPG (mmol/L) |  |  |  |  |  |  |
| Baseline | 9.17 ± 2.70 | 9.64 ± 3.01 | 9.91 ± 2.93 | 0.349 | 0.143 | 0.612 |
| Month 3 | 9.10 ± 2.78 | 9.14 ± 2.61 | 8.12 ± 2.07 | 0.922 | 0.027 | 0.015 |
| Month 6 | 8.91 ± 2.81 | 9.08 ± 2.91 | 7.87 ± 2.07 | 0.737 | 0.019 | 0.007 |
| *P* (month 3 *vs* baseline) | 0.863 | 0.214 | < 0.001 |  |  |  |
| *P* (month 6 *vs* baseline) | 0.564 | 0.244 | < 0.001 |  |  |  |
| *P* (month 6 *vs* month 3) | 0.644 | 0.883 | 0.378 |  |  |  |
| Body weight (kg) |  |  |  |  |  |  |
| Baseline | 69.6 ± 10.0 | 72.3 ± 11.6 | 70.8 ± 11.9 | 0.152 | 0.527 | 0.463 |
| Month 3 | 69.6 ± 9.6 | 72.2 ± 11.9 | 70.9 ± 11.6 | 0.189 | 0.492 | 0.556 |
| Month 6 | 69.4 ± 9.9 | 72.0 ± 11.7 | 71.0 ± 11.6 | 0.180 | 0.411 | 0.625 |
| *P* (month 3 *vs* baseline) | 0.867 | 0.535 | 0.660 |  |  |  |
| *P* (month 6 *vs* baseline) | 0.749 | 0.425 | 0.664 |  |  |  |
| *P* (month 6 *vs* month 3) | 0.407 | 0.738 | 0.836 |  |  |  |
| TC (mmol/L) |  |  |  |  |  |  |
| Baseline | 4.91 ± 1.07 | 4.63 ± 0.93 | 4.97 ± 0.80 | 0.104 | 0.727 | 0.025 |
| Month 3 | 4.77 ± 1.16 | 4.65 ± 1.00 | 4.76 ± 1.09 | 0.514 | 0.942 | 0.548 |
| Month 6 | 4.80 ± 1.10 | 4.54 ± 1.01 | 4.82 ± 1.03 | 0.158 | 0.947 | 0.124 |
| *P* (month 3 *vs* baseline) | 0.213 | 0.818 | 0.062 |  |  |  |
| *P* (month 6 *vs* baseline) | 0.370 | 0.404 | 0.134 |  |  |  |
| *P* (month 6 *vs* month 3) | 0.793 | 0.171 | 0.563 |  |  |  |
| TG (mmol/L) |  |  |  |  |  |  |
| Baseline | 1.69 ± 1.13 | 1.94 ± 1.26 | 2.01 ± 1.48 | 0.236 | 0.123 | 0.635 |
| Month 3 | 1.64 ± 1.20 | 1.78 ± 1.12 | 1.76 ± 1.29 | 0.492 | 0.575 | 0.940 |
| Month 6 | 1.61 ± 1.06 | 1.81 ± 1.10 | 1.70 ± 1.09 | 0.296 | 0.617 | 0.591 |
| *P* (month 3 *vs* baseline) | 0.634 | 0.214 | 0.039 |  |  |  |
| *P* (month 6 *vs* baseline) | 0.396 | 0.249 | 0.008 |  |  |  |
| *P* (month 6 *vs* month 3) | 0.778 | 0.754 | 0.658 |  |  |  |
| HDL-c (mmol/L) |  |  |  |  |  |  |
| Baseline | 1.16 ± 0.31 | 1.05 ± 0.27 | 1.09 ± 0.31 | 0.028 | 0.167 | 0.455 |
| Month 3 | 1.23 ± 0.34 | 1.17 ± 0.28 | 1.19 ± 0.33 | 0.331 | 0.602 | 0.681 |
| Month 6 | 1.20 ± 0.33 | 1.16 ± 0.29 | 1.20 ± 0.36 | 0.511 | 0.905 | 0.451 |
| *P* (month 3 *vs* baseline) | 0.026 | 0.000 | 0.001 |  |  |  |
| *P* (month 6 *vs* baseline) | 0.211 | 0.000 | < 0.001 |  |  |  |
| *P* (month 6 *vs* month 3) | 0.172 | 0.566 | 0.640 |  |  |  |
| LDL-c (mmol/L) |  |  |  |  |  |  |
| Baseline | 3.03 ± 0.96 | 2.75 ± 0.78 | 2.95 ± 0.79 | 0.070 | 0.613 | 0.150 |
| Month 3 | 2.89 ± 1.00 | 2.71 ± 0.81 | 2.83 ± 0.87 | 0.275 | 0.752 | 0.408 |
| Month 6 | 2.81 ± 0.83 | 2.62 ± 0.76 | 2.78 ± 0.86 | 0.190 | 0.844 | 0.277 |
| *P* (month 3 *vs* baseline) | 0.130 | 0.632 | 0.204 |  |  |  |
| *P* (month 6 *vs* baseline) | 0.023 | 0.129 | 0.062 |  |  |  |
| *P* (month 6 *vs* month 3) | 0.406 | 0.176 | 0.431 |  |  |  |

Data are expressed as mean ± SD

Group A: control group; Group B: APP self-management; Group C: APP interactive-management

FPG: fasting plasma glucose; TC: total cholesterol; TG: triglyceride; HDL-c: high-density lipoprotein cholesterol; LDL-c: low-density lipoprotein cholesterol
